# Chinese Herbal Medicine Significantly Impacts the Haematological Variables of the Athlete Biological Passport

**DOI:** 10.3390/ijerph18189533

**Published:** 2021-09-10

**Authors:** William Chih-Wei Chang, Chih-Yuan Wang, Wan-Yi Liu, Chin-Chuan Tsai, Yu-Tse Wu, Mei-Chich Hsu

**Affiliations:** 1 School of Pharmacy, Kaohsiung Medical University, Kaohsiung 807, Taiwan; cwchang@kmu.edu.tw (W.C.-W.C.); s6510624@gmail.com (C.-Y.W.); sh980713@gmail.com (W.-Y.L.); 2 Master Degree Program in Toxicology, Kaohsiung Medical University, Kaohsiung 807, Taiwan; 3 School of Chinese Medicine for Post-Baccalaureate, I-Shou University, Kaohsiung 840, Taiwan; ed103622@edah.org.tw; 4 Chinese Medicine Department, E-Da Hospital, Kaohsiung 824, Taiwan; 5 Drug Development and Value Creation Research Center, Kaohsiung Medical University, Kaohsiung 807, Taiwan; 6 Department of Sports Medicine, Kaohsiung Medical University, Kaohsiung 807, Taiwan; 7 Department of Medical Research, Kaohsiung Medical University Hospital, Kaohsiung 807, Taiwan

**Keywords:** traditional Chinese medicine, supplement, sports drug testing, atypical passport finding, World Anti-Doping Agency

## Abstract

In the fight against sports doping, the Athlete Biological Passport (ABP) system aims to indirectly unveil the doping incidents by monitoring selected biomarkers; however, several unexplored extrinsic factors may dampen a fair interpretation of ABP profiles. Chinese herbal medicine (CHM) plays a pivotal role in the health care system, and some remedies have a long history of being used to treat anaemia. In this study, we addressed the concerns of whether the CHM administration could yield a measurable effect on altering the ABP haematological variables. Forty-eight healthy volunteers were randomly assigned to receive two-week oral administration of one of the six selected CHM products that are commonly prescribed in Taiwan (eight subjects per group). Their blood variables were determined longitudinally in the phases of baseline, intervention, and recovery over 5 weeks. Blood collection and analyses were carried out in strict compliance with relevant operating guidelines. In the groups receiving Angelicae Sinensis Radix, Astragali Radix, and Salviae Miltiorrhizae Radix et Rhizoma, a significant increased reticulocyte percentage and decreased OFF-hr Score were manifested during the intervention, and such effects even sustained for a period of time after withdrawal. All other variables, including haemoglobin and Abnormal Blood Profile Score, did not generate statistical significance. Our results show that the use of CHM may impact the ABP haematological variables. As a consequence, we recommend athletes, particularly those who have been registered in the testing pool, should be aware of taking specific Chinese herbal-based treatment or supplementation, and document any of its usage on the anti-doping forms.

## 1. Introduction

Traditional medicine (TM) has sometimes been portrayed as having a lack of solid evidence for efficacy based on standards used in Western medicine. However, TM is an empirical product that was gathered over centuries of clinical observations and practices [1]. It is undeniable that TM plays a crucial role in the global health care system. Due to the differences in cultural backgrounds, the development and the usage habits of TM somewhat vary between countries [2] and regions [3]. As for Taiwan, TM’s wide-spread popularity has been highlighted by more than 50% of the citizens having received Chinese herbal medicine (CHM) treatment, and this trend of visits has increased over time [4,5]. During the last decades, CHM has been used as ergogenics in sports because scientific data showed that some herbs could potentially enhance muscle strength, improve aerobic endurance, reduce fatigue, or increase mental alertness [6,7]. Nonetheless, CHMs have been less investigated concerning the adverse analytical findings in sports drug testing, possibly because of their complexity in chemical composition, source, and variety. Our knowledge regarding the containing of banned substances is still limited to a handful of CHMs such as steroids in deer musk pod [8], ephedrines in Ephedrae Herba [9], and higenamine in lotus plumule [10]. 

In the fight against sports doping, the World Anti-Doping Agency (WADA) has established the Athlete Biological Passport (ABP) system, aiming to indirectly unveil the doping incidents by monitoring several biological markers rather than detecting the doping substance itself. The markers are repeatedly tested in a longitudinal evaluation of the same athlete, a specific range is built under the consideration of intra-individual variability [11]. The outlier will be flagged as an Atypical Passport Finding (ATPF) and require further investigation. The ABP system is composed of the Steroidal Module and the Haematological Module. The ABP Steroidal Module aims to identify anabolic agents such as testosterone and its precursors. The ABP Haematological Module that examines a panel of haematological markers is employed particularly to identify the use of erythropoiesis-stimulating agents (e.g., recombinant human erythropoietin) or blood transfusion. However, in some cases, physiological variations occur within the ABP, for example, adaptation to altitude, injury, or iron supplementation [12].

On the other hand, CHMs frequently applied to patients for improving the symptoms associated with anaemia, Angelicae Sinensis Radix and Astragali Radix for instance, have been demonstrated to regulate haematopoiesis by a number of previous works [13,14,15]. These CHMs are not included in the prohibited list nor should contain any banned substances. Yet, concerns have been raised about their impact on the ABP haematological variables. In the present study, we administered CHMs including five single herbs and one formula to the healthy volunteers for 2 weeks. Their blood characteristics were determined longitudinally in the phases of baseline, intervention, and recovery during a 5-week period. We then assessed if the CHM administration can yield a measurable effect on altering the ABP biomarkers.

## 2. Materials and Methods

### 2.1. Participants

Forty-eight healthy, physically active adults (24 male aged 25.3 ± 4.3 years and 24 female aged 25.0 ± 4.2 years) volunteered to participate in this study. They are mostly college students who do not play in professional/non-professional sports but stay physically active. Power and sample size calculations were performed using G *Power [16]. We calculated that the total sample size of 48 for one-way ANOVA analysis would provide 80% power with an effect size f of 0.55 to detect differences between the means of six groups at the 5% significance level. The participants were screened to ensure not participating in competitive sporting events; they were not taking any Western or Traditional medicine, herbal supplement, or iron agent; they did not have a history of liver, kidney, lung or heart disease, or alcoholism. The protocol of this study was reviewed and approved by the Institutional Review Board of the E-Da Hospital, Kaohsiung, Taiwan (reference: EMRP21908N). All participants provided written informed consent after receiving an explanation of the study procedures.

### 2.2. Chinese Herbal Medicine

The selection of CHM for this study was based on a domestic survey of CHM in clinical practice for the treatment of anaemia [17]. The five most commonly prescribed single herbs—Angelicae Sinensis Radix (ASR), Astragali Radix (AR), Salviae Miltiorrhizae Radix et Rhizoma (SMR), Asini Corii Colla (ACC), and Spatholobi Caulis (SC)—and one most commonly prescribed formula—Gui-Pi-Tang (GPT)—were employed. The CHM products as concentrated powder were purchased from Sheng Chang Pharmaceutical Company GMP (Taipei, Taiwan), and their detailed information is given in Table S1. These CHM products were manufactured under Good Manufacturing Practice that had undergone authentication, physicochemical examination, traditional extraction, and concentration.

### 2.3. Study Design

**Figure 1:**
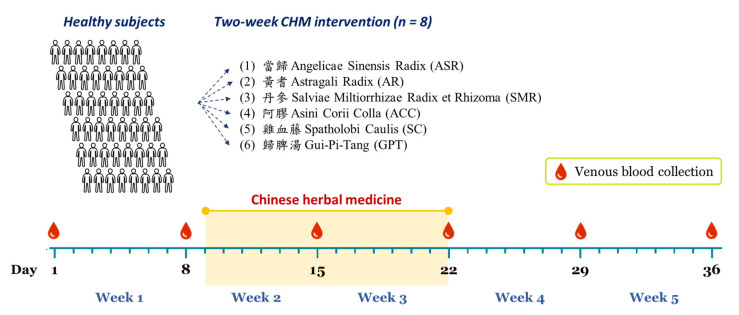
Experimental design of this study.

The study design is depicted in Figure 1 The participants were randomly assigned in a six-arm parallel design to receive oral administration of one of the six CHM products. Each group (*n* = 8) consisted of 4 males and 4 females. The venous blood specimens were collected once a week for a 5-week period. The timeframe of the blood collections was consistent (between 9:00 and 10:00 a.m.). The CHM intervention commenced on day 9 to day 22 (two-week treatment). The dosage regimen of CHM was determined by the maximum dose of recommendation on the labels, 3.6 g per day for the single herbs and 20 g per day for the formula, and such doses were divided twice daily after meals. The participants were not exposed to altitude, hypoxia or other extreme environments (hot or cold). The participants were asked to maintain the usual lifestyle, dietary habit, and physical activity throughout the study period. The blood variables on day 1 and 8 presented the basal values of an individual; day 15 and 22 reflected the impact of CHM intervention; day 29 and 36 showed the recovery phase after CHM withdrawal.

### 2.4. Blood Sampling and Analysis

The blood samples were collected and analysed in accordance with the WADA’s ABP Operating Guidelines [18]. The venous blood specimens were sampled at a study room by experienced medical personnel who maintained a high standard of hygiene. The whole blood sample was collected into a BD Vacutainer^®^ containing K2EDTA (Franklin Lakes, NJ, USA). Afterwards, the sample was kept in a refrigerated state, immediately transported to the laboratory (Health Leader Biomedical Co., Ltd., Kaohsiung, Taiwan) and analysed on the same day of collection. The complete blood counts for each sample were analysed using Sysmex XN9000 (Sysmex Corporation, Kobe, Japan), including red blood cell count (RBC), mean corpuscular volume (MCV), haematocrit (HCT), haemoglobin (HGB), mean corpuscular haemoglobin (MCH), mean corpuscular haemoglobin concentration (MCHC), white blood cell count (WBC), platelet count (PLT), and reticulocyte percentage (RET%). Each blood sample was analysed twice; the differences between the duplicate for HGB should be ≤0.1 g/dL and for RET% should be ≤0.15% (≤1.00% RET) and ≤0.25% (>1.00% RET). If not, the sample was reanalysed until acceptable. Daily instrument check was performed with three different levels of internal QC following the manufacturer’s specifications. 

### 2.5. ABP and Statistical Analyses

Individual OFF-hr Score (OFFS) was calculated as Hb[g/L]–60*√Ret[%] and the Abnormal Blood Profile Score (ABPS) was calculated using an R package [19]. One-way ANOVA with Tukey’s post test was performed using SPSS 22.0 (International Business Machines Corporation, Armonk, NY, USA) for the comparisons of the blood variable between six timepoints within each group. A *p*-value < 0.05 was considered statistically significant. Data are expressed as the mean ± standard deviation.

## 3. Results

**Figure 2:**
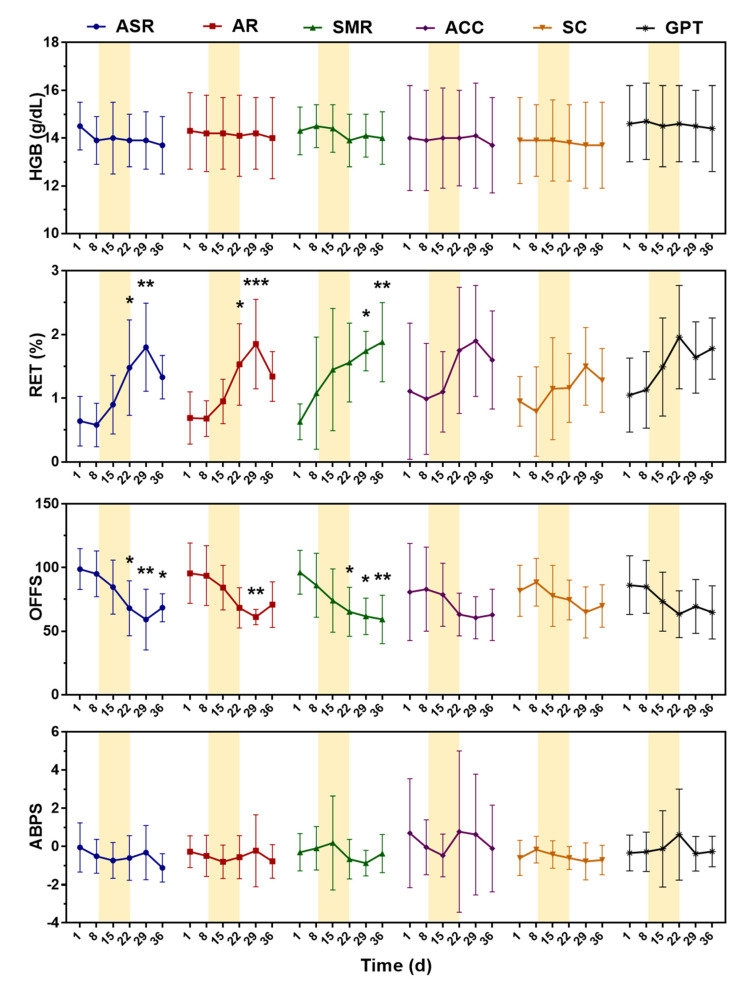
Changes in haemoglobin (HGB), reticulocytes percentage (RET%), OFF-hr Score (OFFS), and Abnormal Blood Profile Score (ABPS) during the administration of Angelicae Sinensis Radix (ASR), Astragali Radix (AR), Salviae Miltiorrhizae Radix et Rhizoma (SMR), Asini Corii Colla (ACC), Spatholobi Caulis (SC), or Gui-Pi-Tang (GPT). Data are expressed as the mean ± standard deviation (*n* = 8). * *p* < 0.050, ** *p* < 0.010, *** *p* < 0.001 as compared with the baseline (day 1) in each independent group using one-way ANOVA with Tukey’s posttest.

The results of primary blood biomarkers of the ABP system during the study period including HGB, RET%, OFFS, and ABPS are depicted in Figure 2. The results of all other measured haematological variables are given in Table 1. Significant differences among the studied timepoints were only found in RET% and OFFS from the groups receiving ASR, AR, and SMR. All other variables did not generate statistical significance. In Figure 2, The colour column manifests the 2-week CHM administration period, from day 9 to day 22. The values on day 1 and 8 are considered as the baseline phase; day 15 and 22 as the intervention phase; day 29 and 36 as the recovery phase. 

In the ASR group, RET% increased from the mean of 0.64% (day 1) to 1.48% (day 22, *p* = 0.028 vs. day 1) and even to 1.80% after withdrawal (day 29, *p* = 0.001 vs. day 1). The OFFS thereby decreased from the mean of 98.8 (day 1) to 68.1 (day 22, *p* = 0.028 vs. day 1), 59.2 (day 29, *p* = 0.002 vs. day 1), and 68.5 (day 36, *p* = 0.031 vs. day 1). 

Similar trends were observed in the AR group. RET% rose from the mean of 0.69% (day 1) to 1.53% (day 22, *p* = 0.016 vs. day 1), and went higher to 1.85% (day 29, *p* < 0.001 vs. day 1). The OFFS declined from the mean of 95.6 (day 1) to 68.4 (day 22, *p* = 0.051 vs. day 1) and 61.1 (day 29, *p* = 0.006 vs. day 1). 

The effects of SMR treatment acting on RET% sustained for a longer time compared with ASR and AR as its value did not drop but maintained at 1.74% (day 29, *p* = 0.020 vs. day 1) and 1.88% (day 36, *p* = 0.006 vs. day 1) in the recovery phase. The baseline OFFS was 96.3 (day 1), and it was decreased to 65.3 (day 22, *p* = 0.044 vs. day 1), 61.7 (day 29, *p* = 0.017 vs. day 1), and 59.3 (day 36, *p* = 0.009 vs. day 1).

In the ACC, SC, and GPT groups, the RET% also seemed to be altered in some of the participants, but overall did not generate a significant difference.

This experiment was designed to collect the samples up to 14 days after the CHMs administration as evaluating the recovery phase. However, the elevated RET% did not completely return to the baseline even at the last collection. The sample collection period should be extended in order to observe the full recovery of RET%.

## 4. Discussion

### 4.1. CHMs on ABP Variables and Haematopoiesis

RETs are the immature non-nucleated red blood cells (RBCs) involved in the process of erythropoiesis. RET maturation begins in the bone marrow and is completed in the blood circulation. An increase in RET is clinically indicative of an increase in red cell production, for example, to overcome the loss of mature RBCs in a haemolytic anaemia [20]. From our results, ASR, AR, and SMR treatments significantly impacted the ABP profile particularly by increasing RET%. Minimal change in other measured haematological variables suggested a blunted response to CHM intervention. Perhaps most of the studies investigating the prospective CHMs were conducted in a blood-deficient experimental model, restored or increased in HGB levels and RBCs thus appeared in the treatment group. However, this phenomenon has not been seen in the healthy subjects of the present study. 

The haematopoietic effect of ASR could be mainly ascribed to the water-soluble fraction because the polysaccharides isolated from ASR were found to restore the HGB levels and promote bone marrow haematopoiesis in different blood-deficient rodent models [14,21] and a clinical case of haemodialysis patient [22]. The underlying mechanisms were demonstrated as the ASR polysaccharides stimulate erythropoietin production and improve iron availability via activating janus kinase 2 (JAK2)/signal transducer and activator of transcription 5 (STAT5) and phosphatidylinositol 3-kinase (PI3K)/protein kinase B (Akt) signalling pathway [13,23,24]. Meanwhile, ASR also ameliorated anaemic condition through several metabolic pathways in a metabolomics analysis [25]. 

AR is another eminent CHM used as adjunctive therapy in clinical practice to promote the haematopoietic function [26]. The main active ingredient is also identified as the water-soluble polysaccharide, and it has been prepared as an injectable dosage form (commercially available) [26] or coupled with iron complexes [27] for facilitating HGB recovery and better treatment. However, ASR and AR are more often prescribed in combination since according to the theory of traditional Chinese medicine, the recipe is able to deliver synergic efficacies by raising the “q” (vital energy) as well as nourishing the “blood” (body circulation) of a person [28]. Studies have shown that combined ASR and AR could stimulate the growth and inhibit the apoptosis of bone marrow stromal cells [15,29]. In addition, the number of RET in peripheral blood was significantly increased, the secretion of haematopoietic growth factor and the proliferation of haematopoietic progenitor cells were promoted in mice receiving the herbs [30,31].

Conversely, SMR’s purported effects to alleviate anaemia have not been evidenced in high-quality human trials. Yet, its ability to increase anticoagulation via inhibiting platelet aggregation is well documented [32]. SMR’s active component tanshinone IIA prevents atherogenesis [33] and salvianolic acid B exhibits high vasodilator potency [34]. In China, SMR is co-extracted with Flos Carthami and prepared as a “Danhong injection” that has been used in the clinical therapy of cardiovascular and cerebrovascular diseases such as myocardial infarction and stroke [35]. 

GPT, a formula of 12 herbs including ASR and AR, is clinically applied to the treatment of blood deficiency. There is no difference in RET% and OFFS after a 2-week GPT administration, possibly due to the relatively low proportion of ASR and AR that made up this formula. Despite the fact that no statistical significance was produced in temporal ABP monitoring of other groups (e.g., ACC, SC, and GPT), it does not guarantee that none of their usages would be flagged as ATPF in real sports drug testing.

### 4.2. ABP and Confounding Factors

The haematological module of ABP is sensitive today to reveal erythropoietin misuse and blood doping. After the administration of recombinant human erythropoietin to healthy volunteers, increased RET% and decreased OFFS were identified, but in some cases followed by increased HGB, RBC, HCT, and MCV, and decreased MCHC depending on the dosing regimens [36,37]. Autologous blood transfusion could be revealed using ABP as the blood withdrawal would stimulate the RET% and impact the OFFS consequently [38]. 

However, the variability of ABP markers can be provoked by natural confounders. Training at altitude reduces individuals’ blood oxygen saturation to a hypoxic state that triggers an erythropoietin mediated increase in haematopoiesis. This natural acclimation to altitude can impact RET%, HGB, and OFFS [39]. Exercise or certain ailment-induced dehydration can cause individuals’ plasma volume to drop. As many ABP variables are dependent on total blood volume, lowered plasma volume through dehydration may cause these levels to appear elevated [40]. Nevertheless, a narrative review [41] argues that the current ABP paradigm is rather robust and the effects of extrinsic factors (e.g., heat acclimation, hypoxic training, or supplementation) still remain difficult to discriminate. 

The misuse of plasma volume expanders (PVE) in sports is prohibited by the WADA. PVE polysaccharides such as dextran, hydroxyethyl starch, and mannitol could control haematological parameters (e.g., HGB and HCT) via the “diluting effect” and could act as masking agents of blood doping [42]. The CHMs are rich in polysaccharides, yet they are not being administered intravenously in the present study as the illicit PVE is normally given. Therefore, the lack of HGB change during the study period should not be ascribed to the polysaccharide-contained CHMs because oral administration may hardly alter the volume of fluid in the circulatory system.

The ABP adopts longitudinal monitoring of variables via a mathematical probabilistic approach (i.e., the adaptive Bayesian model) to indirectly demonstrate the doping use rather than solely rely on the population-based reference intervals [43]. In the forthcoming work, this experiment would be expanded to larger sample size and extended to a longer study period. The future interpretation regarding the impact of CHMs on ABP biomarkers will be tailored to each studied individual with longitudinal evaluations through access to WADA’s Anti-Doping Administration and Management System (ADAMS).

### 4.3. Limitations

We acknowledge certain limitations to this study. Firstly, we do not have a control or placebo group. Even though a placebo effect should not affect the blood formula as the ABP system intends to identify intra-individual abnormal variability over time rather than rely on population reference, a placebo treatment with a cross-over design would help to exclude a potential drift in the measured variables. Secondly, the fact that we combined males and females, two disparate datasets, in a group was not taken into consideration while determining the sample size and would have obscured the final results. Thirdly, herbal medicines are a complex mixture of multiple components but not a single chemical. The exact compositions of the given CHMs are not thoroughly investigated. The current literature still lacks high-quality pharmacological studies and human trials so that remains difficult to provide a clear picture of the mechanism of actions for the CHMs. Finally, the menstruation-related hormonal changes and blood loss in female participants were not adequately monitored. A recent publication [44] assessed the impact of the menstrual cycle on the ABP haematological module in women with regular menses. The authors reported that the ABP markers overall are stable throughout the menstrual cycle but also noted higher levels of RET% in the ovulation and luteal phases as compared to the follicular phase.

## 5. Conclusions

A fair interpretation of ABP profiles is underpinned by a broad understanding of various causes of fluctuations, environmental or medical, for the ABP biomarkers. This study, for the first time, shows that the use of CHM may impact the haematological variables of the ABP. Significantly increased RET% followed by decreased OFFS were demonstrated in the healthy volunteers receiving two-week administration of ASR, AR, and SMR. As CHM is considered a pivotal category of complementary and alternative medicine, we recommend that athletes, particularly those who have been registered in the testing pool, should be aware of taking specific Chinese herbal-based treatment or supplementation, and document any of its usage on the anti-doping forms.

## Figures and Tables

**Table 1:** Changes in the haematological variables other than HGB, RET%, OFFS, and ABPS during the administration of Angelicae Sinensis Radix (ASR), Astragali Radix (AR), Salviae Milti-orrhizae Radix et Rhizoma (SMR), Asini Corii Colla (ACC), Spatholobi Caulis (SC), or Gui-Pi-Tang (GPT). Data are expressed as mean with SD (*n* = 8).

Phase	Baseline				Intervention				Recovery			
Day	1		8		15		22		29		36	
Data	Mean	SD	Mean	SD	Mean	SD	Mean	SD	Mean	SD	Mean	SD
Angelicae Sinensis Radix (ASR)												
RBC (10^6^/μL)	5.25	0.55	5.04	0.51	5.08	0.62	5.07	0.53	5.03	0.50	4.98	0.49
MCV (fL)	86.4	7.3	86.1	7.4	85.6	7.0	85.5	7.1	85.5	7.1	85.5	7.0
HCT (%)	45.1	2.7	43.1	2.3	43.2	3.8	43.1	3.0	42.8	2.9	42.3	2.6
MCH (pg)	27.8	3.1	27.9	3.2	27.8	3.2	27.6	3.1	27.8	3.2	27.7	3.2
MCHC (g/dL)	32.1	1.1	32.3	1.3	32.4	1.3	32.2	1.3	32.4	1.3	32.4	1.4
WBC (/μL)	6055	1036	5931	1193	6041	1558	6181	1513	6608	1157	6283	883
PLT (10^3^/μL)	275.6	76.3	263.1	75.0	261.3	94.5	267.6	80.7	262.0	70.2	269.8	72.0
Astragali Radix (AR)												
RBC (10^6^/μL)	5.06	0.78	5.00	0.71	5.04	0.72	5.00	0.83	5.00	0.74	4.92	0.77
MCV (fL)	87.5	7.4	87.5	7.1	86.7	6.9	87.2	7.1	87.5	7.3	87.7	7.5
HCT (%)	43.9	4.1	43.4	3.6	43.4	3.5	43.1	4.4	43.3	3.9	42.8	4.3
MCH (pg)	28.6	3.1	28.6	3.1	28.4	3.2	28.5	3.2	28.6	3.1	28.7	3.1
MCHC (g/dL)	32.6	1.3	32.7	1.4	32.7	1.6	32.7	1.5	32.7	1.3	32.6	1.3
WBC (/μL)	5896	1392	6361	2120	5929	1440	5881	1445	5783	1365	6216	1314
PLT (10^3^/μL)	282.5	43.7	273.1	26.4	280.5	44.3	279.8	49.2	271.9	64.0	287.3	47.4
Salviae Miltiorrhizae Radix et Rhizoma (SMR)												
RBC (10^6^/μL)	5.11	1.03	5.15	0.92	5.14	0.96	4.97	0.96	4.98	0.91	4.98	0.91
MCV (fL)	89.0	11.0	88.6	11.4	88.3	10.2	88.4	10.9	88.3	11.3	88.9	10.8
HCT (%)	44.5	3.3	44.8	2.5	44.6	3.7	43.1	3.7	43.1	2.5	43.4	2.9
MCH (pg)	28.6	4.1	28.7	3.9	28.6	4.0	28.6	4.1	28.8	4.0	28.8	3.9
MCHC (g/dL)	32.1	1.1	32.3	1.1	32.2	1.3	32.3	1.2	32.6	1.0	32.3	1.0
WBC (/μL)	6286	1264	6105	1934	6353	1788	6456	1960	7104	2367	6130	1998
PLT (10^3^/μL)	255.9	57.0	256.0	49.7	261.1	54.6	254.4	50.5	269.9	64.3	280.5	57.5
Asini Corii Colla (ACC)												
RBC (10^6^/μL)	4.90	0.61	4.87	0.56	4.91	0.63	4.91	0.51	4.99	0.58	4.82	0.51
MCV (fL)	87.6	2.7	87.3	2.6	87.0	2.6	87.4	3.3	87.0	3.0	87.1	3.0
HCT (%)	43.1	6.2	42.5	5.3	42.8	6.0	43.0	5.5	43.4	5.8	42.0	5.4
MCH (pg)	28.4	1.6	28.5	1.7	28.5	1.7	28.5	1.7	28.3	1.7	28.4	1.7
MCHC (g/dL)	32.4	1.0	32.6	1.1	32.6	1.1	32.6	0.9	32.5	1.1	32.6	1.0
WBC (/μL)	7198	2376	6924	2196	6381	2049	6931	2111	7324	2622	6943	2231
PLT (10^3^/μL)	283.6	67.3	282.5	56.8	291.9	63.4	304.9	62.1	291.8	61.7	288.3	59.1
Spatholobi Caulis (SC)												
RBC (10^6^/μL)	4.80	0.59	4.78	0.50	4.78	0.55	4.78	0.54	4.69	0.61	4.69	0.62
MCV (fL)	88.7	3.6	89.0	3.6	87.9	3.0	88.8	3.4	88.2	3.6	88.8	3.8
HCT (%)	42.4	4.4	42.4	4.0	42.0	4.4	42.3	4.0	41.3	4.9	41.5	4.9
MCH (pg)	29.0	1.5	29.0	1.3	29.0	1.5	28.9	1.5	29.2	1.4	29.2	1.5
MCHC (g/dL)	32.7	1.1	32.6	1.0	33.0	1.2	32.5	1.0	33.1	0.9	32.9	1.0
WBC (/μL)	6823	1643	6974	2264	7128	1931	6423	1213	6718	1971	6291	1669
PLT (10^3^/μL)	282.9	83.6	283.1	80.5	275.3	66.6	283.9	79.6	283.1	74.6	286.6	78.7
Gui-Pi-Tang (GPT)												
RBC (10^6^/μL)	5.28	1.08	5.30	1.09	5.25	1.13	5.30	1.14	5.28	1.08	5.21	1.10
MCV (fL)	86.4	8.9	86.4	9.2	86.0	8.5	86.3	9.2	85.9	8.9	86.1	8.9
HCT (%)	44.9	4.7	45.0	4.7	44.4	5.2	44.9	4.7	44.6	4.7	44.2	5.2
MCH (pg)	28.1	3.4	28.2	3.5	28.1	3.3	28.1	3.4	28.1	3.3	28.1	3.4
MCHC (g/dL)	32.5	1.0	32.6	1.3	32.6	1.1	32.5	1.0	32.6	0.9	32.6	1.0
WBC (/μL)	7506	1577	7231	1688	7895	1690	7899	2130	7623	1819	7823	2127
PLT (10^3^/μL)	317.9	76.4	318.3	71.6	315.1	75.8	326.1	62.6	320.4	64.3	329.5	61.3

## Data Availability

Data is contained within the article or Supplementary Material.

## Supplementary Materials

The following are available online at https://www.mdpi.com/article/10.3390/ijerph18189533/s1, Table S1: Information of the Chinese herbal medicine products.
