# Supplementary material for: Chinese Herbal Medicine Significantly Impacts the Haematological Variables of the Athlete Biological Passport
Source: Int J Environ Res Public Health. 2021 Sep 10;18(18):9533. doi: 10.3390/ijerph18189533 (PMC8469363; doi:10.3390/ijerph18189533)
Supplement: Supplementary file 1 [file ijerph-18-09533-s001.zip › ijerph-1354745-supplementary.pdf]

Supporting information

Table S1. Information of the Chinese herbal medicine products.

Product name	Contents	Recommended dose (g/d)	Product photo
Angelicae Sinensis Radix Extract Powder	Angelicae Sinensis Radix 4.44 g<br>The above prepared as the extract 0.67 g<br>Starch 0.33 g	0.8–3.6	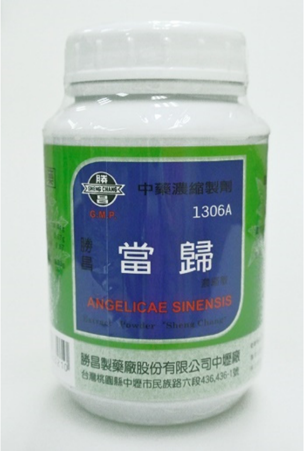
Astragali Radix Extract Powder	Astragali Radix 3.33 g<br>The above prepared as the extract 0.67 g<br>Starch 0.33 g	0.8–3.6	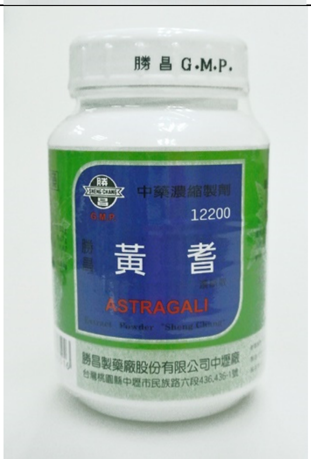
Salviae Miltiorrhizae Radix et Rhizoma Extract Powder	Salviae Miltiorrhizae Radix et Rhizoma 3.47 g<br>The above prepared as the extract 0.67 g<br>Starch 0.33 g	0.8–3.6	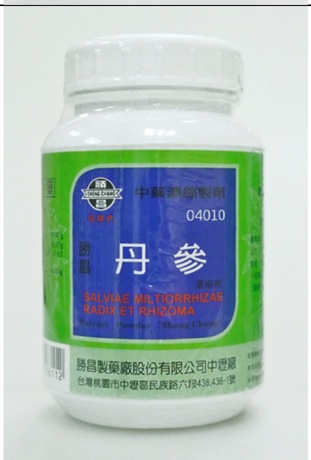

Asini Corii Colla Extract Powder	Asini Corii Colla 0.83<br>The above prepared as the extract 0.5 g<br>Asini Corii Colla 0.2<br>Starch 0.1 g<br>Microcrystalline cellulose 0.2 g	0.8–3.6	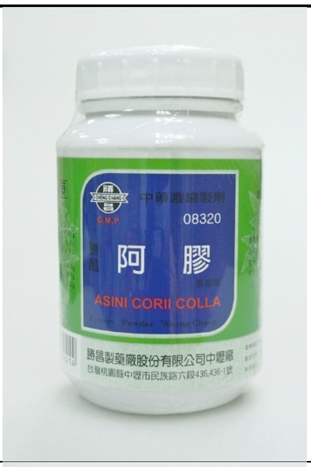
Spatholobi Caulis Extract Powder	Spatholobi Caulis 4.12<br>The above prepared as the extract 0.67 g<br>Starch 0.33 g	0.8–3.6	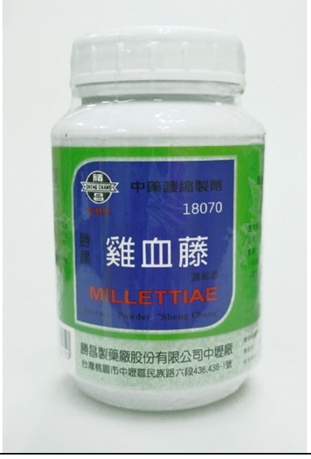
Gui-Pi-Tang	Ginseng Radix 3.0 g<br>Longan Arillus 3.0 g<br>Astragali Radix 3.0 g<br>Glycyrrhizae Radix et Rhizoma Praeparata Cum Melle 1.5 g<br>Atractylodis Macrocephalae Rhizoma 3.0 g<br>Poria 3.0 g<br>Aucklandiae Radix 1.5 g<br>Angelicae Sinensis Radix 3.0 g<br>Ziziphi Spinosae Semen 3.0 g<br>Polygalae Radix 3.0 g<br>Zingiberis Rhizoma Recens 2.0 g<br>Jujubae Fructus 2.0 g<br>The above prepared as the extract 8.5 g<br>Starch 6.5 g	15–20	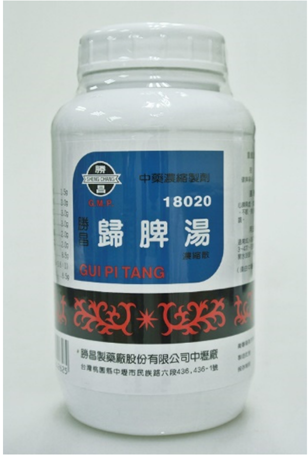
